# Cytotoxic effect of *Alpinia scabra* (Blume) Náves extracts on human breast and ovarian cancer cells

**DOI:** 10.1186/1472-6882-13-314

**Published:** 2013-11-12

**Authors:** Annushuya Subba Reddy, Sri Nurestri Abd Malek, Halijah Ibrahim, Kae Shin Sim

**Affiliations:** 1 Institute of Biological Sciences, Faculty of Science, University of Malaya, Kuala Lumpur 50603, Malaysia

**Keywords:** Zingiberaceae, *Alpinia scabra*, Cytotoxic activity, Ovarian cancer, Breast cancer

## Abstract

**Background:**

*Alpinia scabra*, locally known as 'Lengkuas raya’, is an aromatic, perennial and rhizomatous herb from the family Zingiberaceae. It is a wild species which grows largely on mountains at moderate elevations in Peninsular Malaysia, but it can also survive in the lowlands like in the states of Terengganu and Northern Johor. The present study reports the cytotoxic potential of *A. scabra* extracts from different parts of the plant.

**Methods:**

The experimental approach in the present study was based on a bioassay-guided fractionation. The crude methanol and fractionated extracts (hexane, chloroform and water) from different parts of *A. scabra* (leaves, rhizomes, roots and pseudo stems) were prepared prior to the cytotoxicity evaluation against human ovarian (SKOV-3) and hormone-dependent breast (MCF7) carcinoma cells. The identified cytotoxic extracts were then subjected to chemical investigations in order to identify the active ingredients. A normal human lung fibroblast cell line (MRC-5) was used to determine the specificity for cancerous cells. The cytotoxic extracts and fractions were also subjected to morphological assessment, DNA fragmentation analysis and DAPI nuclear staining.

**Results:**

The leaf (hexane and chloroform) and rhizome (chloroform) extracts showed high inhibitory effect against the tested cells. Ten fractions (LC1-LC10) were yielded after purification of the leaf chloroform extract. Fraction LC4 which showed excellent cytotoxic activity was further purified and resulted in 17 sub-fractions (VLC1-VLC17). Sub-fraction VLC9 showed excellent cytotoxicity against MCF7 and SKOV-3 cells but not toxic against normal MRC-5 cells. Meanwhile, eighteen fractions (RC1-RC18) were obtained after purification of the rhizome chloroform extract, of which fraction RC5 showed cytotoxicity against SKOV-3 cells with high selectivity index. There were marked morphological changes when observed using phase-contrast inverted microscope, DAPI nuclear staining and also DNA fragmentations in MCF7 and SKOV-3 cells after treatment with the cytotoxic extracts and fractions which were indicative of cell apoptosis. Methyl palmitate and methyl stearate were identified in the hexane leaf extract by GC-MS analysis.

**Conclusions:**

The data obtained from the current study demonstrated that the cell death induced by cytotoxic extracts and fractions of *A. scabra* may be due to apoptosis induction which was characterized by apoptotic morphological changes and DNA fragmentation. The active ingredients in the leaf sub-fraction VLC9 and rhizome fraction RC5 may lead to valuable compounds that have the ability to kill cancer cells but not normal cells.

## Background

Up to recently, there is a growing shift of people choosing herbal cures over conventional drugs due to the perception that herbals are safer and with few or no side effects [1]. Reports from the year 1981 to 2002 showed that there are approximately 60% of anticancer agents which are derived from plants and a further 20% are natural product mimics or synthetic compounds derived from natural products [2,3]. Thus, natural products from plants do not only serve as drugs but also provide a rich source of novel structures that may be developed into novel anticancer agents.

Zingiberaceae is one of the largest families in the order Zingiberales which comprises about 1200 species. Zingiberaceae species or commonly known as gingers are distributed throughout the tropics especially in Southeast Asia. There are about 150 species of gingers belonging to 23 genera found in Peninsular Malaysia [4]. Of these, include selected species from the genera *Alpinia, Amomum, Curcuma*, *Kaempferia* and *Zingiber* which have been reported to have medicinal values and have been used for generations in various traditional health care systems. The genus *Alpinia* has been studied widely for its cancer-fighting properties and the chemical substances isolated from *Alpinia* species have been reported to show anticancer activities [5]. In previous study conducted by Lee and Houghton [6], the dichloromethane extracts of *Alpinia officinarum* and *Alpinia galanga* showed strong toxicity towards COR L23 (human non-small cell lung cancer) and MCF7 (human adenocarcinoma). The compound 1’-acetoxychavicol acetate which was isolated from both plants was the major cytotoxic component against COR L23 and MCF7 cancer cells. According to Banjerdpongchai *et al.*[7], 4’-hydroxycinnamaldehyde (4’–HCA) which was isolated from *Alpinia galanga* was cytotoxic to human leukemic HL60 and U937 cell lines in a dose-dependent manner.

*Alpinia scabra*, locally known as 'Lengkuas raya’, is an aromatic, perennial and rhizomatous herb. It is a wild ginger which grows largely on mountains at moderate elevations in Peninsular Malaysia, but it can also survive in the lowlands like in the states of Terengganu and Northern Johor [8]. There is little information available in the literature about *A. scabra* probably due to its limited distribution in the Malesian region. There is only one published scientific report on the cytotoxic activity of leaf and rhizome extracts of *A. scabra*[8]. However, the previous report did not carry out detailed study to evaluate the cytotoxic and apoptotic effects of *A. scabra* on the human cancer cell lines.

The present study was carried out using a bioassay-guided approach to evaluate the cytotoxicity of *A. scabra* extracts from different parts of the plant (leaves, rhizomes, roots and pseudo stems) against human ovarian (SKOV-3) and hormone-dependent breast (MCF7) carcinoma cell lines. MRC-5, a normal human lung fibroblast cell line was used to determine the specificity of the extracts for cancerous cells. In order to evaluate the apoptosis induction capability, all the cytotoxic extracts and fractions were subjected to morphological assessment, DNA fragmentation analysis and DAPI nuclear staining. To our knowledge, this will be the first time such extracts and fractions from the different parts of *A. scabra* are being tested against these cell lines.

## Methods

### Chemicals and reagents

3-(4,5-Dimethylthiazol-2-yl)-2,5-diphenyltetrazolium bromide (MTT), dimethylsulfoxide (DMSO), Doxorubicin, RPMI 1640 medium, Dulbecco’s Modified Eagle’s Medium (DMEM), Minimum Essential Medium (MEM) and 4’,6-Diamidino-2-Phenylindole (DAPI) were obtained from Sigma-Aldrich Company, UK. Methanol, hexane and chloroform were purchased from Merck Company, Germany. Fetal bovine serum (FBS), penicillin, streptomycin and amphotericin B were from PAA Lab, Austria. Suicide Track DNA ladder isolation kit was purchased from Calbiochem, USA.

### Plant sample collection and identification

**Figure 1:**
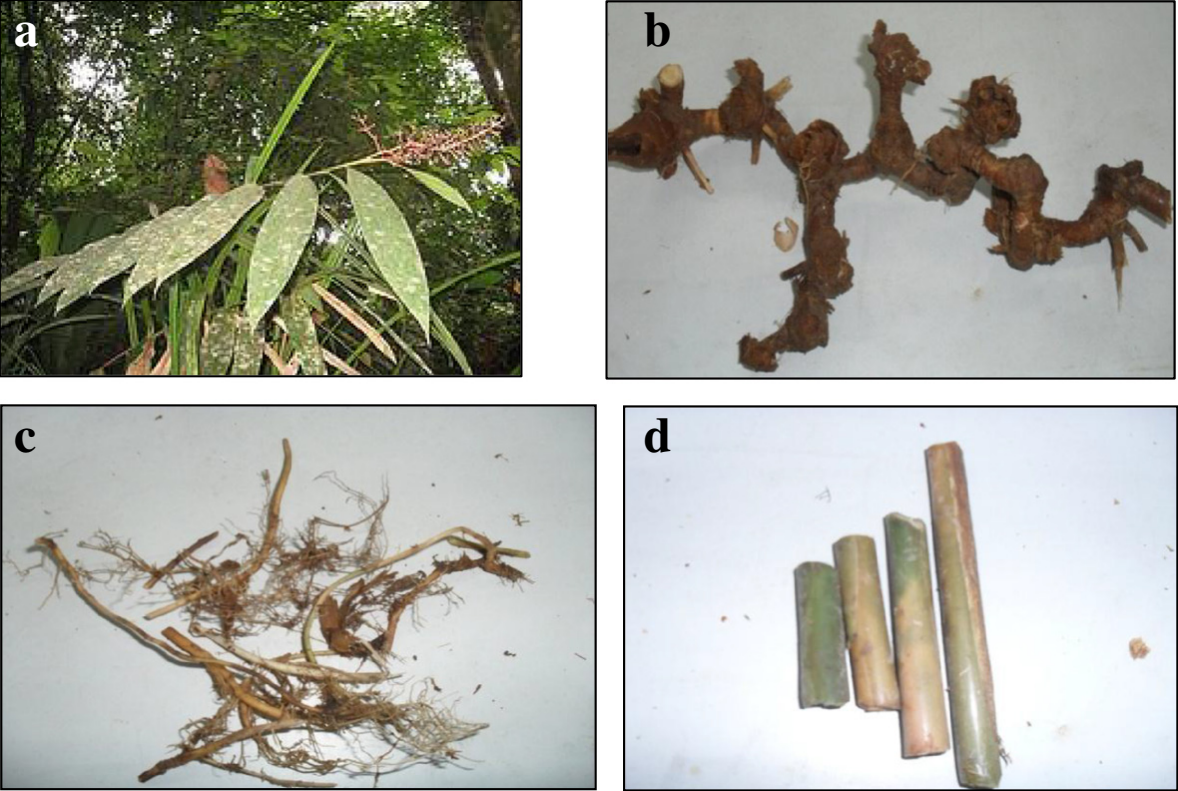
***Alpinia scabra*. (a)** leaves **(b)** rhizomes **(c)** roots and **(d)** pseudo stems.

The fresh leaves, rhizomes, roots and pseudo stems of *A. scabra* were collected from Genting Highland, Pahang, Malaysia. The plant samples were identified by Professor Dr Halijah Ibrahim of Institute of Biological Sciences, Faculty of Science, University of Malaya, Malaysia and a voucher specimen (herbarium no. HI 1419) was deposited at the herbarium of Institute of Biological Sciences, Faculty of Science, University of Malaya, Malaysia. The appearance of *A. scabra* is shown in Figure 1.

### Preparation of extracts

The extracts were prepared as previously described [9] with minor modifications. Briefly, the different parts of *A. scabra* (leaves, rhizomes, roots and pseudo stems) were washed and ground to fine powder. The dried and ground samples were then extracted with 80% methanol for three days at room temperature to obtain the crude methanol extracts. Part of the crude methanol extract was reserved for the cytotoxicity assay while the remaining portions were fractionated with hexane to give the hexane-soluble extracts and hexane-insoluble residues. The hexane-insoluble residues were further partitioned with chloroform and water (1:1, 100 ml: 100 ml) to give the chloroform and water extracts. The weights of the crude methanol and fractionated extracts (hexane, chloroform and water) were measured after solvent evaporation. All the extracts were dissolved in DMSO, except for the water extracts which were dissolved in distilled water to form stock solutions of 20 mg/ml and stored at-20°C before cytotoxicity testing.

### Cell lines and culture medium

The MCF7 (human hormone-dependent breast carcinoma cell line), SKOV-3 (human ovarian cancer cell line) and MRC-5 (human non-cancer lung fibroblast cell line) were purchased from the American Tissue Culture Collection (ATCC, USA). MCF7 cells were maintained in RPMI 1640 medium, SKOV-3 cells in Dulbecco’s Modified Eagle’s Medium (DMEM) and MRC-5 cells maintained in Minimum Essential Medium (MEM), supplemented with 10% fetal bovine serum (FBS), 100 μg/ml penicillin and 50 μg/ml of amphotericin B. The cells were cultured in a 5% CO_2_ incubator (Shel Lab, USA) at 37°C in a humidified atmosphere. The culture was sub-cultured every two to three days and routinely checked under an inverted microscope (Leica Microsystems, Germany) for any contamination.

### MTT assay

The assay was carried out as previously described [9]. Briefly, cells were seeded in a 96-well micro titer plate (Nunc, Denmark) at a concentration of 30,000 cells/ml and incubated in a CO_2_ incubator at 37°C to allow the cells to adhere. After 24 hours, the cells were treated with extracts at six different concentrations, i.e. 1, 10, 25, 50, 75 and 100 μg/ml and incubated for 24, 48 and 72 hours. Cells that were treated with fractions and sub-fractions were incubated at 72 hours only. The cytotoxic activity of each extract and fraction was expressed as IC_50_, which is the concentration of extract (or fraction) that causes 50% inhibition or cell death. Extract (or fraction) with IC_50_ of 20 μg/ml or less is considered active [10]. DMSO was used to dilute the extracts and the final concentration of DMSO in each well was not in excess of 0.5% (v/v). No adverse effect due to presence of DMSO was observed. Doxorubicin was used as positive control.

In the present study, Selectivity Index (SI) of active extract was determined in order to investigate whether the cytotoxic activity was specific to cancer cells. The SI of the extracts is defined as the ratio of cytotoxicity (IC_50_values) on normal lung fibroblast (MRC-5) cells to cancer cells (MCF7 and SKOV-3): SI = IC_50_ on MRC-5 cells/ IC_50_ on cancer cells. Test agents with SI higher than three were considered to have high selectivity towards cancer cells [11]. Three replicate experiments were used to determine the cytotoxicity of each extracts and fractions of *A. scabra.* The IC_50_ values for cytotoxic activity were obtained by non-linear regression using GraphPad Prism statistical software. Data are shown as mean ± SD from three independent experiments.

### Morphological assessment of apoptotic cells by phase-contrast inverted microscope

Cells (3 × 10^4^ cells/ml) in the absence or presence of the active extract (or fraction) at IC_50_ concentrations, were incubated for 24, 48 and (or) 72 hours (based on the time point in which the extract and fraction were active) in 24-well tissue culture plates. At the end of the incubation period, the culture medium was removed and cells were washed with phosphate buffer saline (PBS pH 7.4) and observed under Leica DMI 3000B phase-contrast inverted microscope (Leica Microsystems, Germany) at 200× magnification and photographed.

### DNA fragmentation analysis by agarose electrophoresis

Detection of apoptotic fragmented DNA was performed using the Suicide-Track DNA isolation kit (Calbiochem, USA) according to manufacturer’s protocol. Briefly, MCF7 and SKOV-3 cells were cultured and treated with cytotoxic extracts, fractions and doxorubicin (positive control) for 24 hours according to the IC_50_ values. After 24 hours of incubation, the floating and trypsinized-adherant of treated and untreated cells were collected and total DNA was extracted from the cells. Samples were loaded into a 1.5% agarose gel and separated by electrophoresis. DNA fragments were stained with 0.5 μg/ml ethidium bromide and were visualized under UV illumination using a Gene Flash gel documentation system (Syngene Bio imaging, UK).

### Morphological detection of apoptosis using DAPI nuclear stain

The occurrence of apoptosis in MCF7 and SKOV-3 cells was evaluated using 4’6-diamidino-2-phenylindole (DAPI, Sigma) staining. Cells (1 × 10^6^) were plated onto 6-well tissue culture plate and incubated in a CO_2_ incubator at 37°C for 24 hours. After 24 hours, the cells were treated with cytotoxic extracts, fractions, sub-fraction and doxorubicin (positive control) for 24 hours at concentration corresponding to the IC_50_ values. Negative control comprised of cells not treated with any extract. After the incubated period, the cells were then harvested and washed with PBS. The resulting cell pellet was fixed with acetone at -20°C for 30 min. The cells were then stained with DAPI solution (1 μg/ml) at 4°C for 30 minutes. Stained cells were spotted onto a slide and cover slips were then mounted onto glass microscope slides and observed under fluorescence microscopy (Olympus BX51) using a 358 nm excitation and 460 nm emission fluorescent filter.

### Bioassay-guided fractionation of leaf chloroform extract

The cytotoxic chloroform extract of leaves (9.0 g) was subjected to vacuum liquid chromatography (VLC) which is a rapid crude fractionation system. The column (7 cm diameter, 30 cm length) was packed with 540.0 g of silica gel 60 (Merck, 0.063-0.200 mm) as stationary phase. The elution of components present in the extract were started with chloroform and then the polarity of the eluent was gradually increased with addition of methanol and finally with only methanol. Elution was monitored by thin layer chromatography (TLC) and the eluent vials were pooled together based on similar pattern of TLC spots into a total of 10 fractions labelled as LC1 to LC10. All the 10 fractions were then tested for cytotoxicity against MCF7, SKOV-3 and MRC-5 cell lines using MTT assay at 72 hours. Among the 10 fractions, only fraction LC4 was found to be active in the cytotoxicity screening against MCF7 and SKOV-3 cell lines. Thus, LC4 was selected for further isolation and purification work.

**Figure 2:**
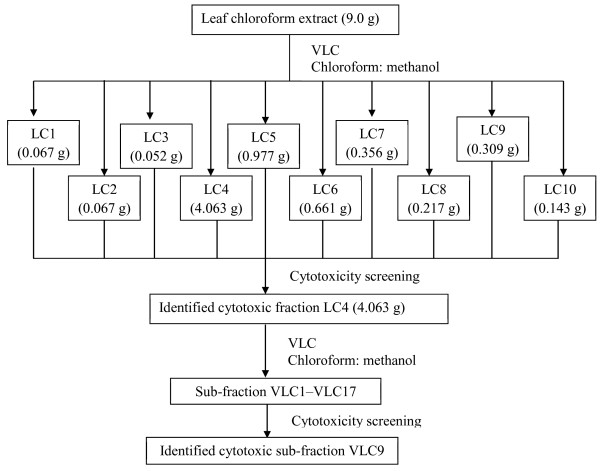
Flow chart of bioassay-guided fractionation of cytotoxic leaf chloroform extract.

The cytotoxic fraction LC4 which was a dark brown paste was then purified using VLC. The column was packed with 60.0 g of silica gel 60 (Merck, 0.063-0.200 mm) as the stationary phase and the ratio of the fraction to silica gel was 1:60. In brief, elution of components in the fraction started with chloroform and then its polarity was gradually increased with addition of methanol and finally with methanol. Elution of components from the column was monitored by TLC and eluent vials with similar pattern of TLC profile were combined to give 17 sub-fractions (VLC1 to VLC17). Some of the sub-fractions (weight more than 20 mg) were then tested for cytotoxicity against MCF7, SKOV–3 and MRC-5 cell lines using MTT assay at 72 hours. A flow chart of the bioassay-guided fractionation of the cytotoxic leaf chloroform extractis shown in Figure 2.

### Bioassay-guided fractionation of rhizome chloroform extract

**Figure 3:**
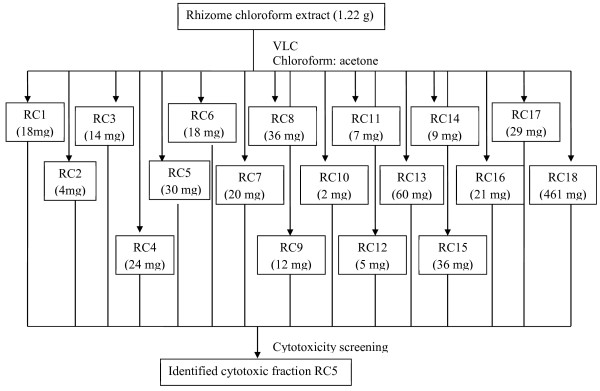
Flow chart of bioassay-guided fractionation of cytotoxic rhizome chloroform extract.

The chloroform extract of rhizomes (2.0 g) was subjected to a rapid crude fractionation using VLC. The column (7 cm diameter, 30 cm length) was packed with 120.0 g of silica gel (Merck, 0.063-0.200 mm) as the stationary phase and the ratio of the extract to silica gel was 1:60. In brief, the components in the fraction were initially eluted with chloroform and then its polarity was gradually increased with addition of acetone and finally methanol. Elution of components from the column was monitored by TLC. Eluent vials with similar pattern of TLC profile were combined to give 18 fractions (RC1 to RC18). All the 18 fractions were then tested for cytotoxicity against SKOV-3 and MRC-5 cells. Figure 3 shows the flow chart of the bioassay-guided fractionation of the rhizome chloroform extract.

### GC-MS analysis

GC-MS analysis was performed using an Agilent Technologies 6980 N gas chromatograph equipped with a 5975 Mass Selective Detector (70 eV direct inlet) as described by Malek *et al.*[12] with some modifications; the HP-5 ms capillary column (5% phenylmethylsiloxane) with column dimensions 30.0 m × 25 mm × 25 um was initially set at 100°C, then the temperature of the oven was increased at 5°C per minute to 300°C and hold for 10 minutes using helium as carrier gas at flow rate 1 ml/min. The total ion chromatogram obtained was auto integrated by ChemStation and the components were identified by comparison with an accompanying mass spectral database [13]. Only mass spectral fragmentation pattern that gave greater than 90% match were accepted.

## Results and discussion

### Extraction yield of *A. scabra* extracts

Solvent extraction is the most popular method used in plant sample preparation. The crude methanol extracts were firstly prepared and further fractionated into hexane, chloroform and water extracts as a single solvent may not be enough to identify certain extracts responsible for the activity. The yield of methanol extracts from different parts of *A. scabra* is shown in Table 1, whereas the yield of extracts fractionated from crude methanol extracts is shown in Table 2. The percentage of crude methanol extract yield was based on the weight of dried and ground plant materials and the percentage yield of fractionated extracts was based on the weight of crude methanol extract used.

**Table 1:** **Yield of the methanol extracts from different parts of *A. scabra***

**Plant part**	**Sample/Extract**	**Weight (g) (%)**
Leaves	Fresh samples	1000.00
	Dried and ground plant material	600.00 (60.00)
	Methanol extract	41.22 (6.87)
Rhizomes	Fresh samples	7000.00
	Dried and ground plant material	400.00 (5.71)
	Methanol extract	17.86 (4.47)
Roots	Fresh samples	900.00
	Dried and ground plant material	100.00 (10.00)
	Methanol extract	7.11 (7.11)
Pseudo stems	Fresh samples	8000.00
	Dried and ground plant material	650.00 (8.13)
	Methanol extract	47.38 (7.29)

**Table 2:** Yield of extracts fractionated from the crude methanol extracts

**Plant part**	**Extract**	**Yield of extracts (g)**	**Percentage (%)**
Leaves	Hexane	0.50	1.21
(extracted from 41.22 g of methanol extract)	Chloroform	9.64	23.39
	Water	21.50	52.16
Rhizomes	Hexane	0.65	3.62
(extracted from 17.86 g of methanol extract)	Chloroform	1.22	6.83
	Water	11.99	67.12
Roots	Hexane	0.31	4.36
(extracted from 7.11 g of methanol extract)	Chloroform	0.63	8.86
	Water	5.14	72.29
Pseudo stems	Hexane	0.48	1.01
(extracted from 47.38 g of methanol extract)	Chloroform	3.23	6.82
	Water	33.05	69.76

### Cytotoxic activities of *A. scabra* extracts

In the present study, the cytotoxic effect (IC_50_) of the crude methanol and fractionated extracts (hexane, chloroform and water) from the different parts of *A. scabra* were investigated on two human cancer cells (MCF7 and SKOV-3) and one normal non-cancer cells (MRC-5) using MTT assay. MTT assay is commonly used in cell biology for the study of growth factor, cytokines and cytotoxicity of chemotherapeutic agents as it offers a quantitative and simple method for evaluating cell population’s response to external factors. Doxorubicin was used as the positive control in the present study because it is a commonly used chemotherapeutic drug for the treatment of acute leukemia, lymphomas and different types of solid tumours such as breast, liver and lung cancers [14]. Cytotoxic activity (IC_50_) and Selectivity Index (SI) of the extracts of leaves, rhizomes, roots and pseudo stems of *A. scabra* are summarized in Tables 3, 4, 5 and 6, respectively.

**Table 3:** **Cytotoxic activity (IC**_**50**_**) of *A. scabra *** **leaf extracts**

**Extracts**	**Treatment duration (hour)**	**IC**_**50**_^**a**^**(μg/ml) (SI**^**b**^**)**		
		**MCF7**	**SKOV-3**	**MRC-5**
Methanol	24	90.67 ± 6.11	47.00 ± 11.53	>100
	48	56.27 ± 9.18	37.67 ± 2.52	57.58 ± 1.25
	72	53.33 ± 6.43	34.33 ± 0.58	65.35 ± 1.68
Hexane	24	**19.30 ± 5.70 (1.6)**	**18.00 ± 2.65 (1.7)**	31.38 ± 2.31
	48	**15.30 ± 4.04 (1.0)**	**6.00 ± 1.00 (2.4)**	**14.63 ± 2.08**
	72	**16.33 ± 0.58 (1.0)**	**4.93 ± 0.12 (3.2)**	**15.90 ± 0.94**
Chloroform	24	**18.80 ± 1.06 (2.4)**	**20.00 ± 1.00 (2.3)**	45.88 ± 3.81
	48	23.67 ± 7.64	**14.33 ± 1.53 (2.3)**	32.26 ± 2.11
	72	25.00 ± 0.00	**14.67 ± 0.58 (2.2)**	32.90 ± 0.76
Water	24	>100	>100	>100
	48	>100	>100	>100
	72	>100	>100	>100
Doxorubicin^c^	24	**2.50 ± 0.10**	**2.50 ± 0.10**	>100
	48	**2.37 ± 0.23 (32.4)**	**1.77 ± 0.15 (43.4)**	76.87 ± 12.64
	72	**0.39 ± 0.02 (4.3)**	**1.47 ± 0.15 (1.1)**	**1.68 ± 0.42**

^a^Data are presented as mean ± SD from three independent experiments triplicate for each. Values in bold characters are considered to have cytotoxic activity (IC_50_20 μg/ml or less); ^b^SelectivityIndex (SI); ^c^Positive control.

**Table 4:** **Cytotoxic activity (IC**_**50 **_**μg/ml) of *A. scabra *** **rhizome extracts**

**Extracts**	**Treatment duration (hour)**	**IC**_**50**_^**a**^**(μg/ml) (SI**^**b**^**)**		
		**MCF7**	**SKOV-3**	**MRC-5**
Methanol	24	>100	>100	>100
	48	>100	>100	>100
	72	>100	>100	>100
Hexane	24	79.67 ± 10.60	40.00 ± 2.00	73.69 ± 2.23
	48	60.00 ± 5.66	25.67 ± 0.58	41.31 ± 2.26
	72	57.33 ± 1.15	24.00 ± 3.46	53.49 ± 2.71
Chloroform	24	70.67 ± 23.12	21.67 ± 4.73	78.08 ± 7.76
	48	39.00 ± 1.41	**19.33 ± 0.58 (2.3)**	43.56 ± 0.54
	72	37.67 ± 0.58	**17.33 ± 0.58 (2.6)**	44.65 ± 2.57
Water	24	>100	>100	>100
	48	>100	>100	>100
	72	>100	>100	>100
Doxorubicin^c^	24	**2.50 ± 0.10**	**2.50 ± 0.10**	>100
	48	**2.37 ± 0.23 (32.4)**	**1.77 ± 0.15 (43.4)**	76.87 ± 12.64
	72	**0.39 ± 0.02 (4.3)**	**1.47 ± 0.15 (1.1)**	**1.68 ± 0.42**

^a^Data are presented as mean ± SD from three independent experiments triplicate for each. Values in bold characters are considered to have cytotoxic activity (IC_50_20 μg/ml or less); ^b^Selectivity Index (SI); ^c^Positive control.

**Table 5:** **Cytotoxic activity (IC**_**50 **_**μg/ml) of *A. scabra *** **root extracts**

**Extracts**	**Treatment duration (hour)**	**IC**_**50**_^**a**^**(μg/ml) (SI**^**b**^**)**		
		**MCF7**	**SKOV-3**	**MRC-5**
Methanol	24	70.67 ± 5.86	56.67 ± 1.15	44.98 ± 10.59
	48	47.67 ± 4.93	34.67 ± 0.58	47.70 ± 8.68
	72	64.00 ± 0.00	34.00 ± 4.36	38.94 ± 5.02
Hexane	24	56.00 ± 2.00	33.33 ± 1.15	30.59 ± 0.36
	48	33.67 ± 5.03	28.33 ± 3.51	30.71 ± 3.15
	72	40.67 ± 1.15	28.00 ± 1.73	29.59 ± 5.98
Chloroform	24	67. 33 ± 5.03	44.00 ± 5.29	30.27 ± 0.33
	48	37.33 ± 4.16	37.00 ± 0.58	51.09 ± 10.80
	72	42.67 ± 2.31	33.67 ± 3.21	38.39 ± 5.57
Water	24	>100	>100	>100
	48	>100	>100	>100
	72	>100	>100	>100
Doxorubicin^c^	24	**2.50 ± 0.10**	**2.50 ± 0.10**	>100
	48	**2.37 ± 0.23 (32.4)**	**1.77 ± 0.15 (43.4)**	76.87 ± 12.64
	72	**0.39 ± 0.02 (4.3)**	**1.47 ± 0.15 (1.1)**	**1.68 ± 0.42**

^a^Data are presented as mean ± SD from three independent experiments triplicate for each. Values in bold characters are considered to have cytotoxic activity (IC_50_20 μg/ml or less); ^b^Selectivity Index (SI); ^c^Positive control.

**Table 6:** **Cytotoxic activity (IC**_**50 **_**μg/ml) of *A. scabra *** **pseudo stem extracts**

**Extracts**	**Treatment duration (hour)**	**IC**_**50**_^**a **^**(μg/ml) (SI**^**b**^**)**		
		**MCF7**	**SKOV-3**	**MRC-5**
Methanol	24	>100	>100	>100
	48	>100	>100	>100
	72	>100	>100	>100
Hexane	24	>100	40.67 ± 3.51	>100
	48	84.00 ± 1.00	34.00 ± 1.73	55.86 ± 16.71
	72	67.30 ± 1.15	34.67 ± 0.58	49.74 ± 1.33
Chloroform	24	80.00 ± 15.10	56.00 ± 5.29	>100
	48	66.67 ± 1.16	30.67 ± 5.13	52.86 ± 1.75
	72	59.30 ± 1.15	33.00 ± 1.73	48.18 ± 2.34
Water	24	>100	>100	>100
	48	>100	>100	>100
	72	>100	>100	>100
Doxorubicin^c^	24	**2.50 ± 0.10**	**2.50 ± 0.10**	>100
	48	**2.37 ± 0.23 (32.4)**	**1.77 ± 0.15 (43.4)**	76.87 ± 12.64
	72	**0.39 ± 0.02 (4.3)**	**1.47 ± 0.15 (1.1)**	**1.68 ± 0.42**

^a^Data are presented as mean ± SD from three independent experiments triplicate for each. Values in bold characters are considered to have cytotoxic activity (IC_50_20 μg/ml or less); ^b^Selectivity Index (SI); ^c^Positive control.

Generally, the root and pseudo stem extracts displayed weaker cytotoxicity profile against all the tested human cell lines compared to the leaf and rhizome extracts. Among the extracts, hexane and chloroform extracts showed better cytotoxic activity against all the tested cell lines compared to the methanol and water extracts. The hexane and chloroform extracts of leaves demonstrated active cytotoxic effect against MCF7 and SKOV-3 cells (Table 3). The leaf hexane extract showed high inhibition against MCF7 cells with IC_50_ value of 15.30 μg/ml at 48 hours, in comparison to IC_50_ values of 19.30 and 16.33 μg/ml at 24 and 72 hours, respectively. It is interesting to note that the chloroform extract of leaves only demonstrated active cytotoxicity against MCF7 cells at 24 hours (18.8 μg/ml), but not at 48 and 72 hours.

The hexane and chloroform extracts of leaves and the chloroform extract of rhizomes displayed active cytotoxic effect against the SKOV-3 cells with IC_50_ values in decreasing trend from 24 to 72 hours. This indicates that the longer the incubation time of the extracts in cells, the better the cytotoxicity results. The hexane extract of leaves showed an excellent inhibition towards SKOV-3 cells with IC_50_ value of 4.93 μg/ml at 72 hours, in comparison to IC_50_ values of 18.00 and 6.00 μg/ml at 24 and 48 hours, respectively. The chloroform extract of leaves possessed the strongest cytotoxicity at 48 hours with IC_50_ of 14.33 μg/ml compared to IC_50_ values at 24 and 72 hours. Meanwhile, the chloroform extract from the rhizomes showed high inhibition towards SKOV-3 cells with IC_50_ value of 17.30 μg/ml at 72 hours, in comparison to IC_50_ values of 21.67 and 19.33 μg/ml at 24 and 48 hours, respectively.

MRC-5 cell line has been used as a normal cell model in many similar studies [9,15-17]. In the current study, all the four samples (leaves, rhizomes, roots and pseudostems) were tested against MRC-5 normal cells and only the leaf hexane extract showed cytotoxicity at 48 and 72 hours with the IC_50_ values of 14.64 and 15.90 μg/ml, respectively. Selectivity of the active extracts were determined but none of the active extracts showed selectivity to the cancer cells as all the selectivity indexes were lower than three, except for the leaf hexane extract which showed selectivity towards SKOV-3 cells at 72 hours with the SI value of 3.2 (Table 3).

The leaf (hexane and chloroform) and rhizome (chloroform) extracts were selected for the bioassay-guided fractionation as these extracts showed strong cytotoxic effect against the selected cancer cells (IC_50_ values of 20 μg/ml or less).

### Bioassay-guided fractionation of the leaf chloroform extract

The ten fractions (LC1-LC10) obtained from VLC were tested for cytotoxicity against MCF7, SKOV-3 and MRC-5 cell lines using MTT assay. The fraction LC4 was the only fraction found to be active in the cytotoxicity screening against MCF7 and SKOV-3 cell lines with IC_50_values of 18.53 and 11.12 μg/ml, respectively (Table 7). Hence, fraction LC4 was warranted for further purification by VLC and yielded 17 sub-fractions (VLC1-VLC17; Figure 2). As shown in Table 7, sub-fraction VLC 9 showed good cytotoxicity against MCF7 and SKOV-3 cell lines (IC_50_ values of 15.53 and 10.89 μg/ml, respectively) but weak cytotoxicity profile against the MRC-5 cell line (IC_50_value >100 μg/ml). As shown in Table 7, the cytotoxicity in ascending order was leaf chloroform extract < LC 4 < VLC9. This may be due to the cytotoxic compounds which present in VLC9 after the purification of the leaf chloroform extract and LC4 *via* VLC. Thus, the active ingredients in VLC9 may lead to valuable compounds that have the ability to kill cancer cells but not toxic against normal MRC-5 cells.

**Table 7:** **Cytotoxic activity (IC**_**50 **_**μg/ml) of fractions and sub-fraction obtained from leaf chloroform extract**

**Fraction (LC)/Sub-fraction (VLC)**	**IC**_**50**_^**a**^**(μg/ml)**		**MRC-5**
	**MCF7**	**SKOV-3**	
LC1	50.00 ± 5.00	92.30 ± 4.78	>100
LC2	42.20 ± 4.48	56.00 ± 6.60	>100
LC3	33.40 ± 4.05	40.50 ± 2.56	>100
**LC4**	**18.53 ± 1.02**	**11.12 ± 0.24**	>100
LC5	51.18 ± 4.56	39.18 ± 1.34	>100
LC6	>100	>100	>100
LC7	>100	>100	>100
LC8	>100	>100	>100
LC9	>100	>100	>100
LC10	>100	>100	>100
**VLC9**	**15.53 ± 0.50**	**10.89 ± 0.64**	>100
Chloroform	25.00 ± 0.00	14.67 ± 0.58	32.90 ± 0.76

^a^Data are presented as mean ± SD from three independent experiments triplicate for each. Values in bold characters are considered to have cytotoxic activity (IC_50_20 μg/ml or less).

### Bioassay-guided fractionation of the rhizome chloroform extract

The cytotoxic effect of the fractions (RC1–RC18) derived from the chloroform extract of rhizome by VLC was evaluated in order to determine the fraction that give the highest activity. Table 8 shows the IC_50_ values of the 18 fractions from the chloroform extract of rhizome. Fraction RC5 was the only fraction which exhibited remarkable cytotoxicity (IC_50_ value of 2.84 μg/ml) and showed high selectivity (SI value of 14.15) against the SKOV-3 cells, compared to the rhizome chloroform extract. This exhibited the improvement of cytotoxicity and selectivity after the purification procedure.

**Table 8:** **Cytotoxic activity (IC**_**50 **_**μg/ml) of fractions obtained from the rhizome chloroform extract**

**Fraction**	**IC**_**50**_^**a**^**(μg/ml)(SI**^**b**^**)**	
	**SKOV-3**	**MRC-5**
RC1	94.56 ± 4.32	95.70 ± 3.00
RC2	88.90 ± 4.78	66.00 ± 5. 66
RC3	25.49 ± 2.56	50.80 ± 1.24
RC4	23.35 ± 2.24	71.00 ± 3.40
**RC5**	**2.84 ± 0.60 (14.15)**	40.20 ± 1.50
RC6	39.90 ± 1.18	>100
RC7	32.63 ± 6.78	>100
RC8	50.24 ± 7.78	>100
RC9	64.50 ± 1.40	>100
RC10	66.82 ± 2.26	>100
RC11	51.30 ± 3.12	>100
RC12	45.70 ± 4.00	>100
RC13	21.60 ± 10.80	>100
RC14	29.70 ± 2.50	>100
RC15	25.33 ± 3.56	>100
RC16	24.75 ± 2.85	>100
RC17	45.08 ± 3.45	>100
RC18	43.89 ± 4.76	>100
Chloroform	17.33 ± 0.58 (2.6)	44.65 ± 2.57

^a^Data are presented as mean ± SD from three independent experiments triplicate for each. Values in bold characters are considered to have cytotoxic activity (IC_50_20 μg/ml or less); ^b^Selectivity Index (SI).

### GC-MS analysis of leaf hexane extract

The cytotoxic hexane leaf extracts were analysed using GC-MS in the present study. The identified compounds are 61.02% methyl palmitate and 24.91% methyl stearate which comprise of 85.93% of the total hexane extract. Methyl palmitate EI-MS m/z (%): 270 [M] ^+^ (2), 239 (2), 227 (5), 213 (2), 199 (4), 185 (5), 171 (8), 157 (2), 143 (18), 129 (8), 115 (4), 107 (1), 97 (8), 87 (70), 74 (100), 65 (1), 55 (30). Methyl stearate EI-MS m/z (%): 298 [M] ^+^ (6), 255 (8), 241 (2), 213 (4), 199 (8), 185 (6), 143 (26), 129 (20), 111 (4), 97 (10), 87 (70), 74 (100), 55(56). All the compounds were identified by GC-MS analysis as well as comparison of its mass spectral data with reported data [18].

Previous report by Sri Nurestri *et al*. [18] suggested that methyl esters might exert cytotoxic effect against normal MRC-5 cells but not on KB, MCF7 and HCT116 cells. This finding supports the data from the present study on the cytotoxicity of *A. scabra* extracts against MRC-5 cells. As shown in Table 3, the hexane leaf extract showed cytotoxic activity on MRC-5 cells at 48 and 72 hours with IC_50_ values of 14.63 and 15.90 μg/ml, respectively. This may be due to the presence of methyl palmitate and methyl stearate in the extract. Furthermore, this finding on cytotoxicity of methyl esters is supported by Takeara *et al*. [19] which reported that methyl palmitate showed cytotoxic effect on T-cell leukemia cell line (Molt-4) with IC_50_ value of 2.28 μg/ml while methyl stearate was cytotoxic to acute promyeloblastic leukemia cell line (HL-60) and Molt-4 cell line with IC_50_ values of 3.08 and 4.65 μg/ml, respectively.

### Morphological assessment of apoptotic cells by phase-contrast inverted microscope

**Figure 4:**
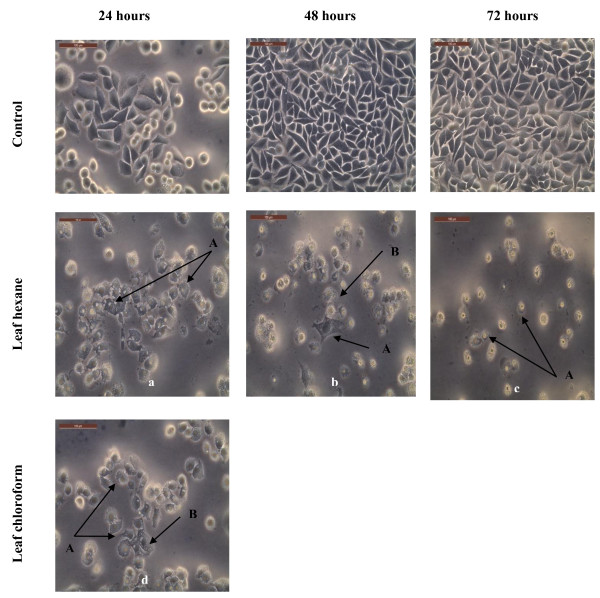
**Morphological observation of MCF7 cells treated with the cytotoxic leaf hexane and chloroform extracts under phase-contrast inverted microscope (magnification 200×).** Arrows indicate (A) cell shrinkage and (B) membrane blebbing as evidence of apoptosis. Note that the cells were treated with the following concentrations of extracts: **a** = 19.3 μg/ml, **b** = 15.30 μg/ml, **c** = 16.33 μg/ml and **d** = 18.80 μg/ml. Figures shown were obtained from at least three independent experiments with similar parameter.

**Figure 5:**
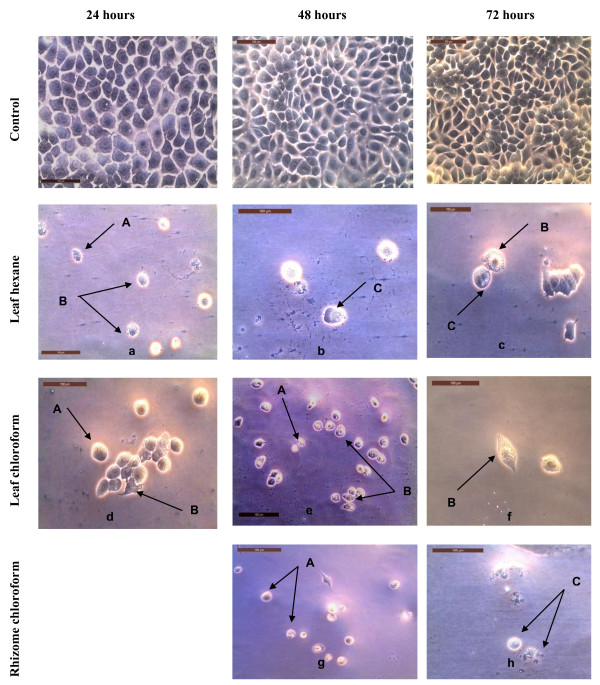
**Morphological observation of SKOV-3 cells treated with the cytotoxic leaf hexane, leaf chloroform and rhizome chloroform extracts under phase-contrast inverted microscope (magnification 200×).** Arrows indicate (A) cell shrinkage; (B) membrane blebbing and (C) apoptotic bodies as evidence of apoptosis. Note that the cells were treated with the following concentrations: **a** = 18.00 μg/ml, **b** = 6.00 μg/ml, **c** = 4.93 μg/ml, **d** = 20.00 μg/ml, **e** = 14.33 μg/ml, **f** = 14.67 μg/ml, **g** = 19.33 μg/ml and **h** = 17.33 μg/ml. Figures shown were obtained from at least three independent experiments with similar parameter.

**Figure 6:**
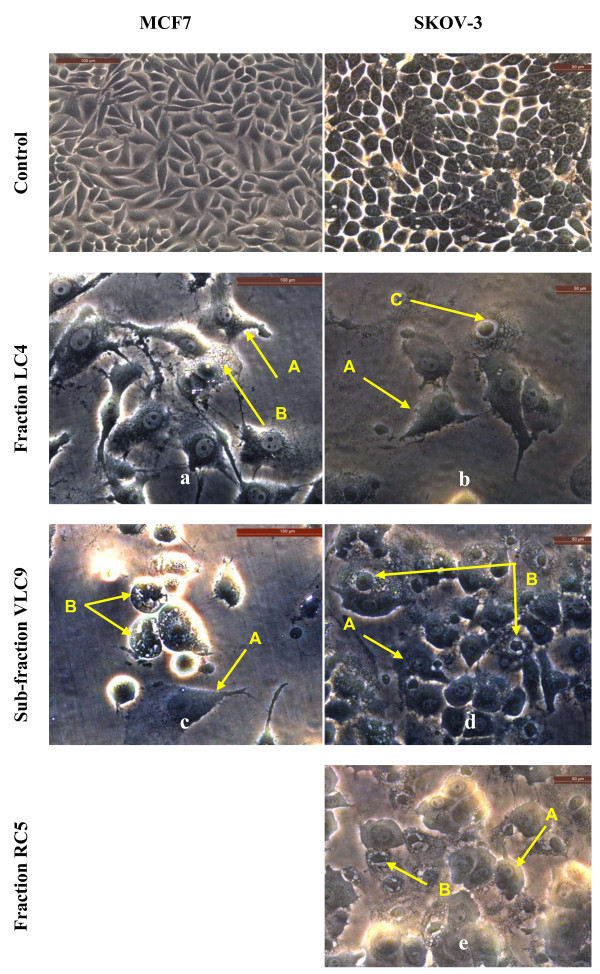
**Morphological observation of MCF7 and SKOV-3 cells treated with the cytotoxic fractions LC4, VLC9 and RC5 under phase-contrast inverted microscope (magnification 200×).** Arrows indicate (A) cell shrinkage; (B) membrane blebbing and (C) apoptotic bodies as evidence of apoptosis. Note that the cells were treated with the following concentrations: **a** = 18.53 μg/ml, **b** = 11.12 μg/ml, **c** = 15.53 μg/ml, **d** = 10.89 μg/ml and **e** = 2.84 μg/ml. Figures shown were obtained from at least three independent experiments with similar parameter.

The results from the present study (Figures 4, 5 and 6) showed that there were obvious morphological changes in MCF7 and SKOV-3 cells after treatment with the cytotoxic extracts, fractions and sub-fraction which were indicative of cell apoptosis. The untreated control MCF7 and SKOV-3 cells maintained their original morphology which are cuboids and in polygonal shapes, and were adherent to the plates. The MCF7 cells were treated with hexane and chloroform extracts of leaves (Figure 4) while SKOV-3 cells were treated with leaf hexane, leaf chloroform and rhizome chloroform extracts (Figure 5) for 24, 48 and (or) 72 hours according to the IC_50_ values (Tables 3 and 4). Figure 6 shows MCF7 and SKOV-3 cells treated with fraction LC4, sub-fraction VLC9 and fraction RC5 at 72 hours according to the IC_50_ values in Tables 7 and 8. The most recognizable morphological features of apoptotic cells observed in this study were shrinkage of cells due to cytoplasmic condensation, rounding up of cells, bleb formation, chromatin condensation and apoptotic bodies’ formation. The other morphological change observed in apoptotic cells was the rounded up cells losing contact with neighbouring cells and caused some sensitive cells to detach from the surface of the well plates. This morphological observation of apoptotic cells were in agreement with previous report [15].

### DNA fragmentation analysis by agarose electrophoresis

**Figure 7:**
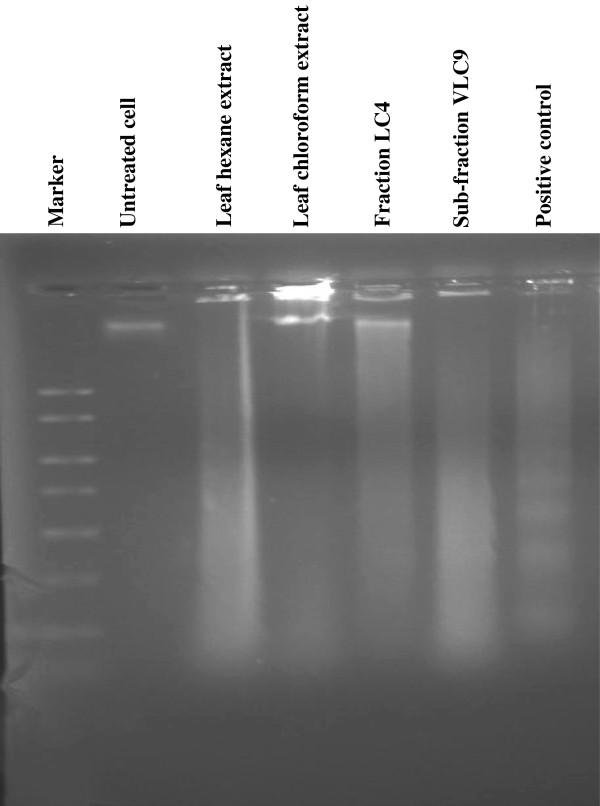
DNA fragmentation of MCF7 cells after treated with cytotoxic extracts and sub-fractions for 24 hours.

**Figure 8:**
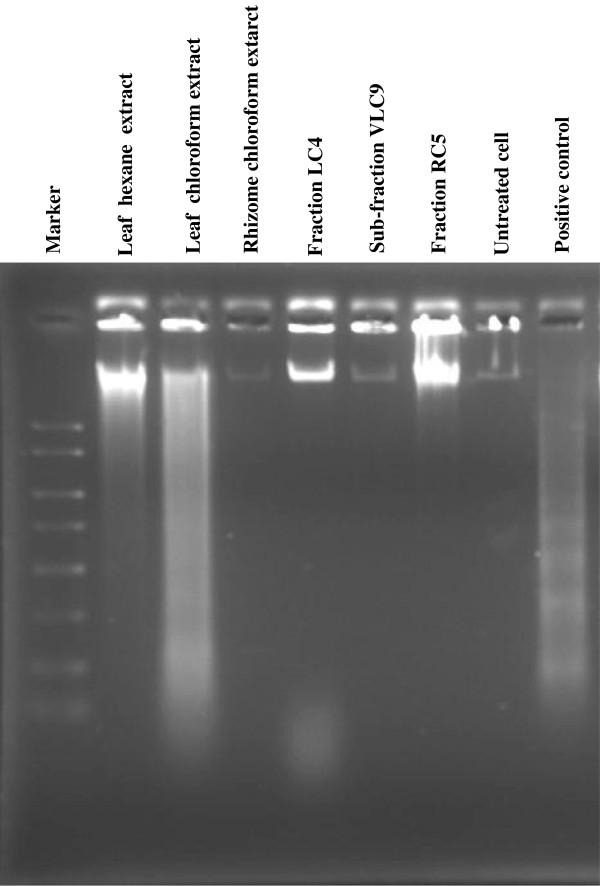
DNA fragmentation of SKOV-3 cells after treated with cytotoxic active extracts and sub-fractions for 24 hours.

DNA fragmentation is a biochemical hallmark of apoptotic cell death. To elucidate whether the active extracts and fractions decrease cell survival by the induction of DNA fragmentation, genomic DNA isolated from MCF7 and SKOV-3 cells were exposed according to the IC_50_ value concentration, electrophoresed and photographed as shown in Figures 7 and 8. Typical DNA ladder formation can be seen clearly in the MCF7 and SKOV-3 cells treated with doxorubicin (positive control) whereas DNA from untreated MCF7 and SKOV-3 cells did not show any fragmentation or smearing. In MCF7 cells treated with cytotoxic active extracts and sub-fractions (Figure 7), the formation of DNA ladder was observed less clearly as there were interspersing smear in the lanes. This pattern was noticeably observed in MCF7 cells treated with hexane and chloroform extract of leaf, fraction LC4 and sub-fraction VLC9 at concentrations of 19.30, 18.80, 18.53 and 15.53 μg/ml, respectively. The smearing could be due to some post-apoptotic necrosis cells [15].

For the SKOV-3 cells (Figure 8), the ladder-like appearance of DNA observed mildly in the cells treated with hexane and chloroform extract of leaf at concentrations of 18.0 and 20.0 μg/ml, respectively. SKOV-3 cells which were treated with chloroform extract of rhizome, fraction LC4, sub-fraction VLC9 and fraction RC5 at concentrations of 21.67, 11.12, 10.89 and 2.84 μg/ml, respectively did not show any DNA laddering or even smearing effect. This could be due to the concentration of the particular extract and sub-fractions which were used to treat the cells were low since at lower doses of treatment, only high molecular weight intact DNA was observed [20]. Besides that, in some cases, DNA fragmentation appears to be delayed, partial, or absent in cells which otherwise meet the morphological criteria for apoptosis and maybe show more limited DNA degradation with the formation of 300-or 50-kb fragments [21]. The large band present at the top of the gel as observed in SKOV-3 cells treated with fraction LC4 and RC5 may represent large semi-fragmented pieces of DNA and indicates incomplete apoptotic fragmentation in the sample material [22].

### Morphological detection of apoptosis using DAPI nuclear stain

**Figure 9:**
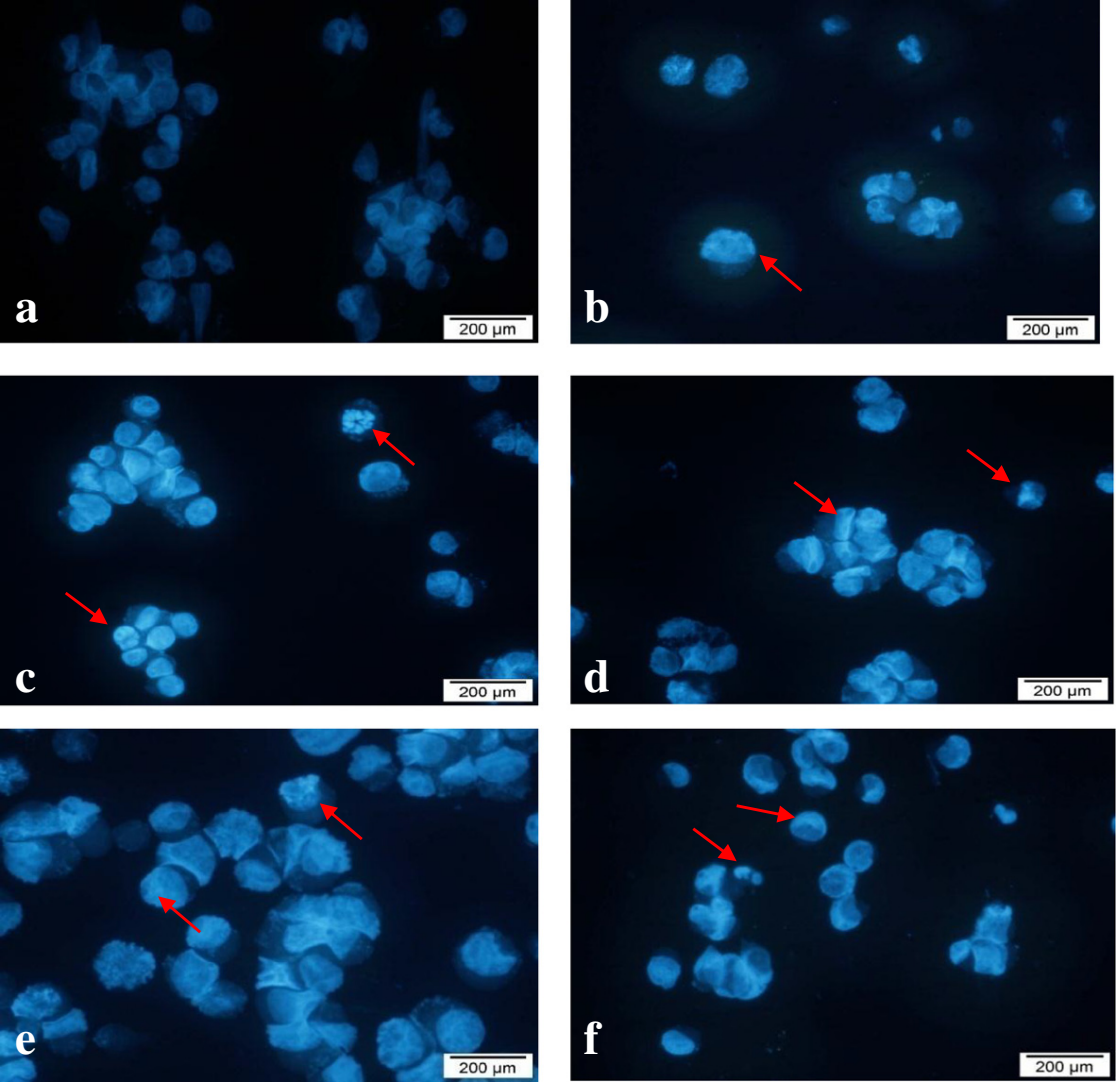
**Morphological observation with DAPI staining by fluorescence microscope for MCF7 cells at 24 hours (magnification 400×).** Arrows indicate signs of nuclear shrinkage and chromatin condensation. DNA samples in the untreated cells were homogenously stained and less intense compared to those in treated cells. Note that the cells were treated with the following extracts/sub-fractions: **a** = control, **b** = leaf hexane, **c** = leaf chloroform, **d** = fraction LC4, **e** = Sub-fraction VLC9 and **f** = positive control. Figures shown were obtained from at least three independent experiments with similar parameter.

**Figure 10:**
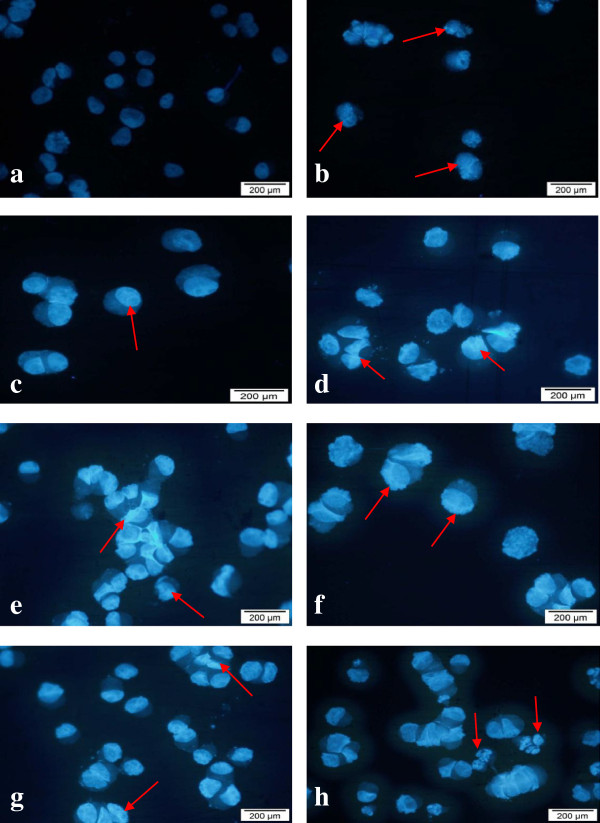
**Morphological observation with DAPI staining by fluorescence microscope for SKOV-3 cells at 24 hours (magnification 400×).** Arrows indicate signs of nuclear shrinkage and chromatin condensation. The untreated cells were rounded in shape and the large nuclei were homogenously stained with less bright blue color. Note that the cells were treated with the following extracts/sub-fractions: **a** = control, **b** = leaf hexane, **c** = leaf chloroform, **d** = rhizome chloroform, **e** = fraction LC4, **f** = Sub-fraction VLC9, **g** = fraction RC5 and **h** = positive control. Figures shown were obtained from at least three independent experiments with similar parameter.

DAPI is a fluorescent stain that allows examination of nuclei in a fluorescence microscope for morphological assessment of changes during apoptosis [23]. Apoptosis is initially characterized by morphological features, such as chromatin condensation, nuclear fragmentation and membrane blebbing [24]. To gain an insight on the effect of cytotoxic extracts, fractions, sub-fraction and doxorubicin on nuclear alterations, cells were stained with DAPI. Figures 9 and 10 show the apoptotic morphological characteristics, as visualized by DAPI staining, of MCF7 and SKOV-3 cells treated (for 24 hours) with cytotoxic extracts, fractions, sub-fraction and doxorubicin (positive control) according to the IC_50_ values. In the control-untreated group [Figures 9(a) and 10(a)], the cells were rounded in shape and the large nuclei were homogenously stained with a less bright blue colour. This is because when healthy cells are exposed to DAPI, staining is restricted to chromatin. Treated MCF7 and SKOV-3 cells displayed bright blue fluorescence with higher intensity than untreated cells due to the highly condensed chromatin.

Besides that, signs of nuclear shrinkage and chromatin condensation which are hallmark of apoptosis were also observed as shown in Figures 9 and 10. Apoptosis is also characterized by the condensation of nuclear chromatin followed by the eventual breakup of the chromatin leading to nuclear fragmentation [25]. This indicated that MCF7 and SKOV-3 cells underwent apoptosis when treated with the cytotoxic extracts, fractions and sub-fraction. This nuclear morphological changes were in agreement with previous report [26].

## Conclusions

The data obtained from the current study provide some preliminary information on the cytotoxic effect of *A. scabra*. Cell death induced by the cytotoxic extracts and fractions may be due to apoptosis which were characterized by apoptotic morphological changes, DNA fragmentations and DAPI nuclear staining. Investigations of cell cycle analysis and the status of well-established apoptotic markers are now underway, in order to provide more convincing evidence of apoptosis induction. Further studies on the isolation and identification of pure compounds from the identified cytotoxic active fractions (VLC9 and RC5) are now in progress.

## Competing interests

The authors declare that they have no competing interests.

## Authors’ contributions

ASR performed the experiments, analyzed the data and wrote the manuscript. SNAM evaluated the data, supervised the work and edited the manuscript. HI determined the species to be studied, identified and partially collected the plant, propose the concept of the study and edited the manuscript. SKS designed the current project, supervised the work and edited the manuscript. All authors have read and approved the final manuscript.

## Pre-publication history

The pre-publication history for this paper can be accessed here:

http://www.biomedcentral.com/1472-6882/13/314/prepub
